# Supplementary figures and images for: Integrated Transcriptome Analysis Identified Key Expansin Genes Associated with Wheat Cell Wall, Grain Weight and Yield
Source: Plants (Basel). 2023 Aug 4;12(15):2868. doi: 10.3390/plants12152868 (PMC10421294; doi:10.3390/plants12152868)

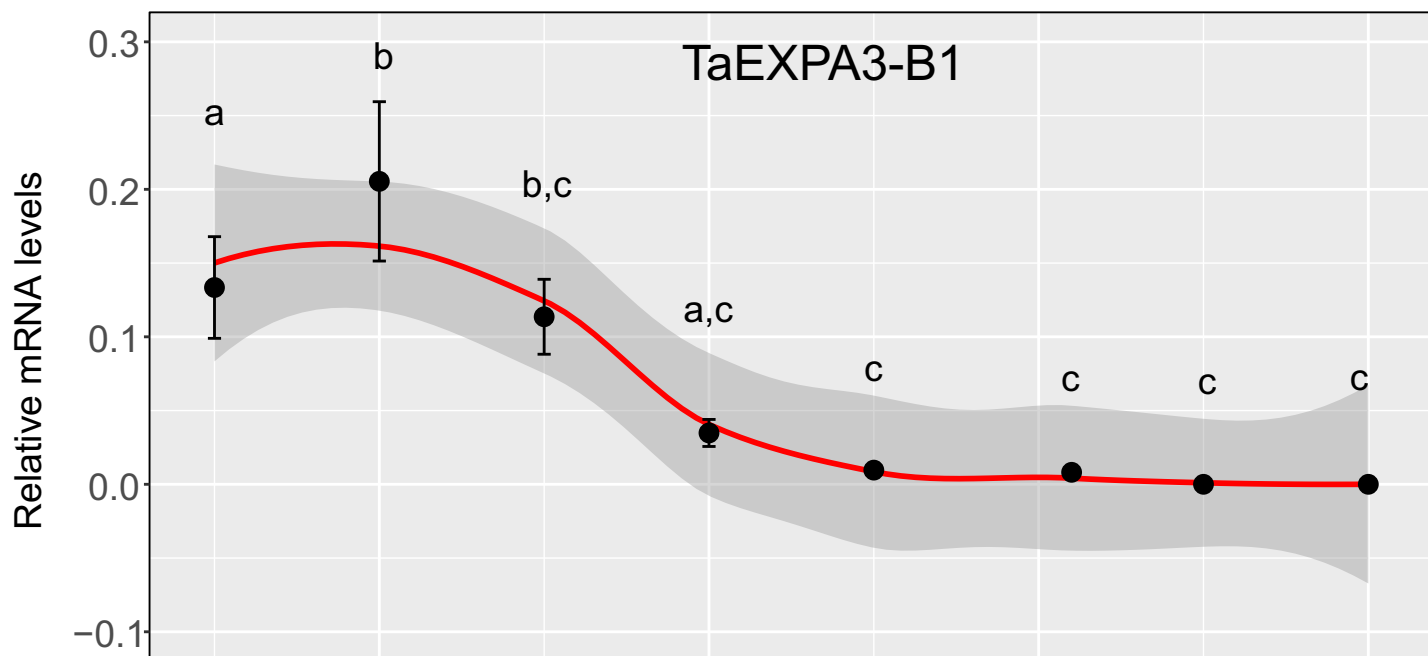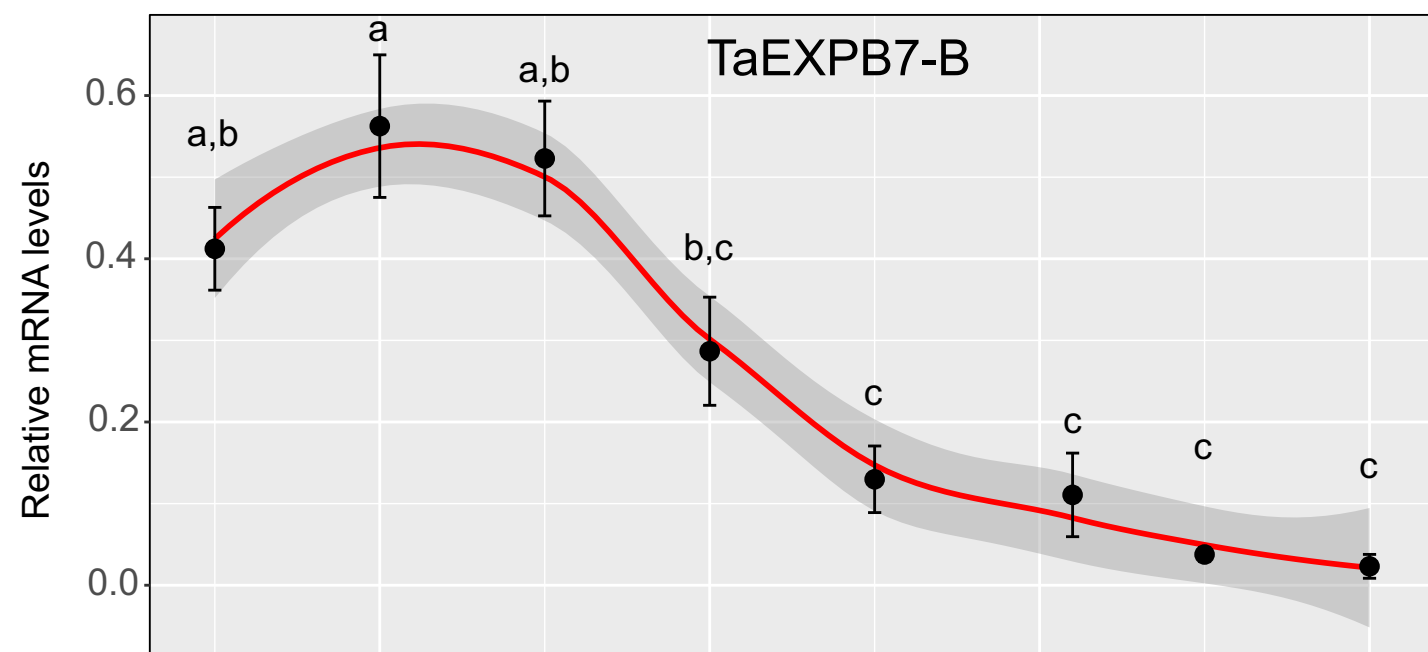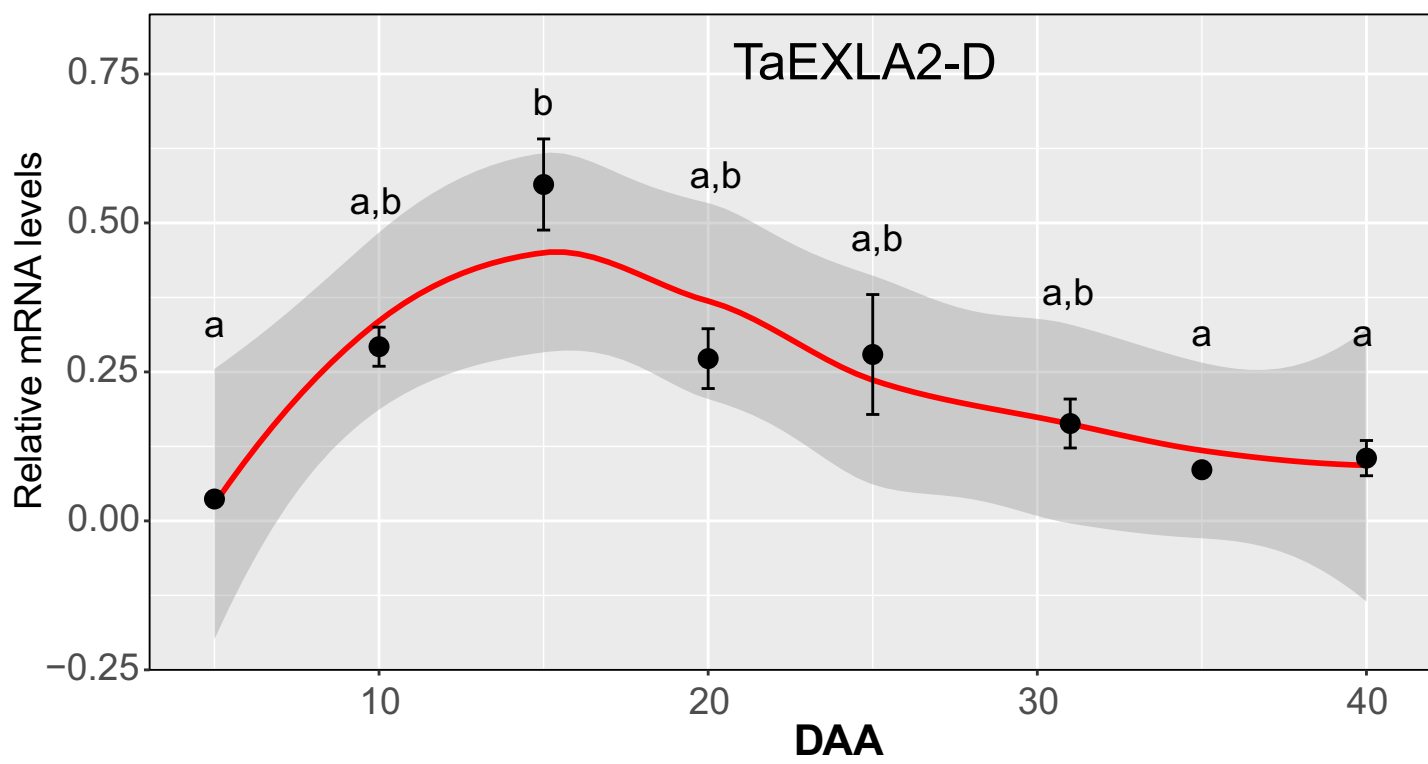

Supplement: Supplementary file 1 [file plants-12-02868-s001.zip › supplementary material/Supplementary FigureS1.pdf]

TaEXPA3-B1

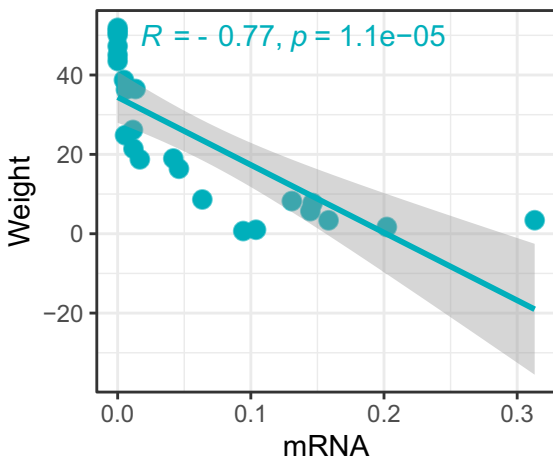

TaEXPB7-B

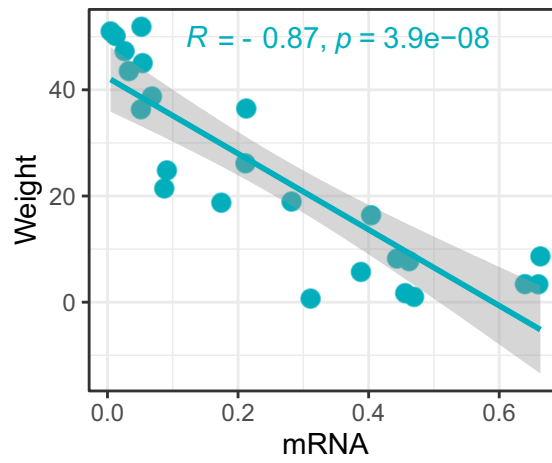

TaEXLA3-B

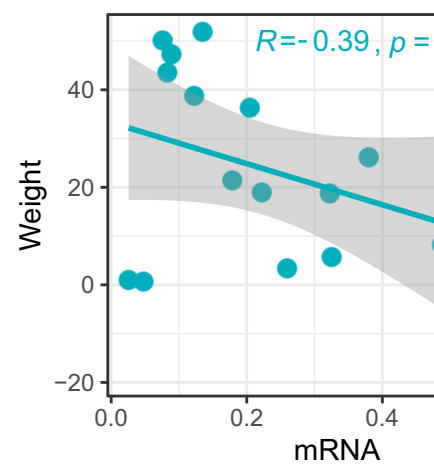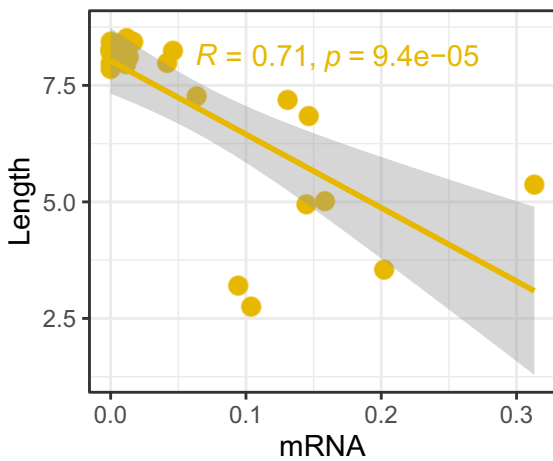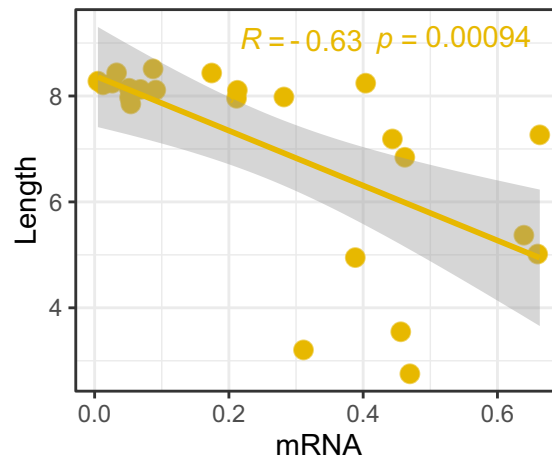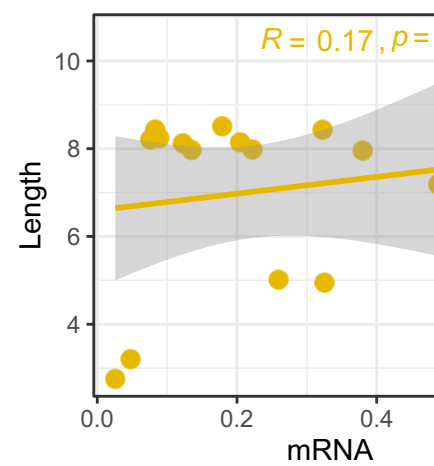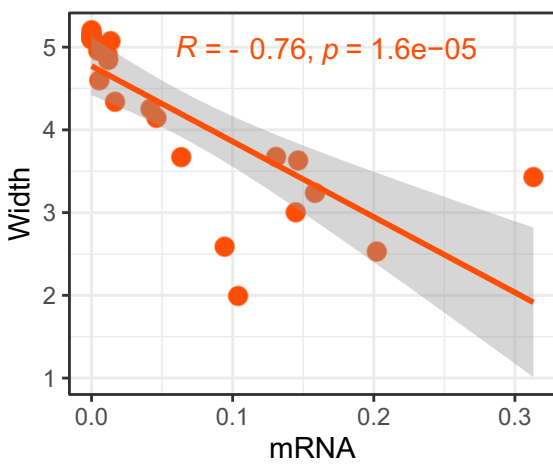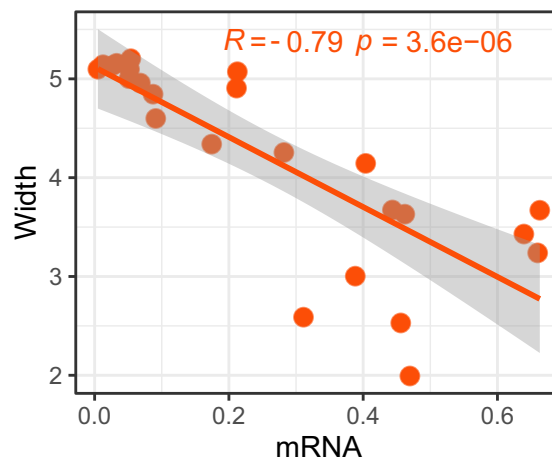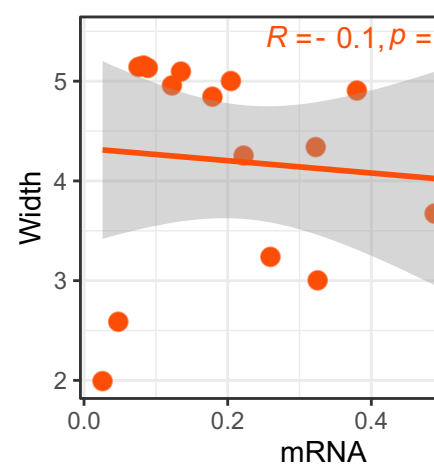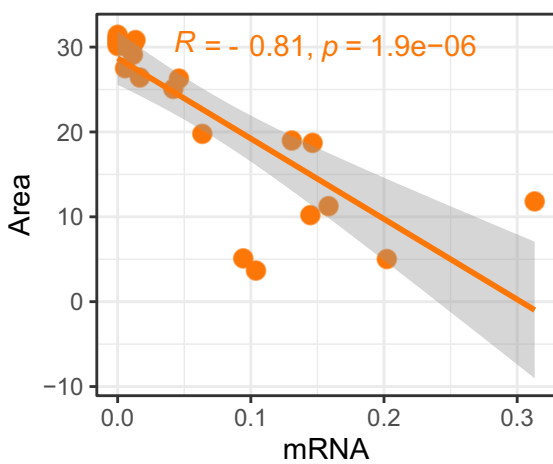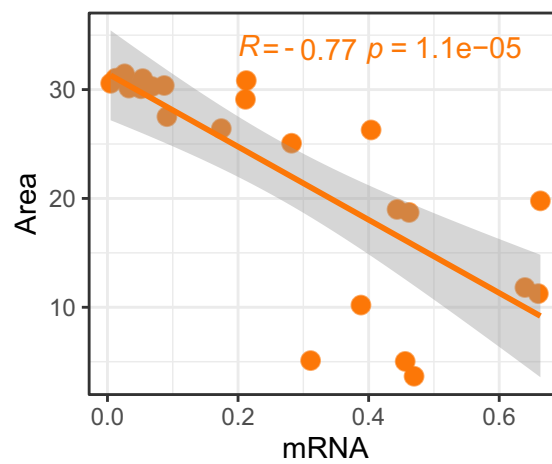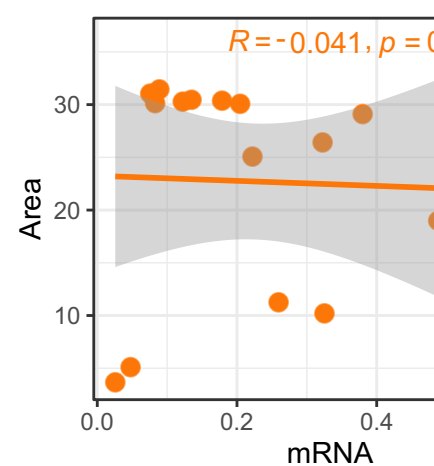

Supplement: Supplementary file 1 [file plants-12-02868-s001.zip › supplementary material/Supplementary FigureS2.pdf]
